# Abscopal effect-induced spontaneous regression of distant metastases in malignant mesenchymal tumor: a case report

**DOI:** 10.3389/fonc.2024.1475129

**Published:** 2024-11-27

**Authors:** Meltem Kirli Bolukbas, Cemile Ozdemir, Esengul Kocak Uzel

**Affiliations:** ^1^ Department of Radiation Oncology, Health Sciences University Bakirkoy Dr. Sadi Konuk Training and Research Hospital, Istanbul, Türkiye; ^2^ Department of Pathology, Bahcelievler Public Hospital, Istanbul, Türkiye

**Keywords:** malignant mesenchymal tumor, abscopal effect, case report, metastatic disease, radiotherapy

## Abstract

The abscopal effect refers to an anti-tumor response that occurs in areas where radiotherapy (RT) has not been directly administered but is triggered by the immune system. We presented a case of an undifferentiated pleomorphic sarcoma with three relapses that showed a complete response after distant metastatic disease. The tumor was initially detected in the left pectoral muscle. Fifteen months after adjuvant RT and chemotherapy, a nearby recurrent lesion was surgically removed. Another 15 months later, a second recurrence appeared on the left lateral chest wall. The patient underwent a third surgery and received adjuvant radiation, but distant metastases were discovered 6 months later. Shortly after a biopsy confirmed distant metastasis, all metastatic foci went into spontaneous remission. This phenomenon is identified as the abscopal effect. The patient experienced no metastasis or local recurrences during follow-up and showed a complete response to the abscopal effect for 36 months. The abscopal effect in malignant mesenchymal tumors is extremely rare.

## Introduction

The abscopal effect is the term used to describe the anti-tumor response that develops in the area not treated with radiotherapy (RT) when radiation activates the immune response. The abscopal effect is a very rare condition and was first described by Mole in 1953 after realizing the unexpected shrinkage of lesions outside the radiation field (1). It is often reported in malignant melanoma, known as an immunogenic cancer (2–4). Apart from this, other cancer types in which the abscopal effect is defined include breast cancer, lymphoma, lung cancer, cervical cancer, renal cell cancer, and hepatocellular cancer (5–10).

A systematic review of the literature between 1969 and 2014 identified only 46 reported cases of abscopal responses (11). The abscopal effect is extremely rare in malignant mesenchymal tumors and has been described in only a few cases in the literature (12–14). In this article, we present a case of a 50-year-old male patient with an undifferentiated pleomorphic sarcoma with three relapses showing a complete response after a biopsy proved distant metastatic disease.

## Case history

**Figure 1:**
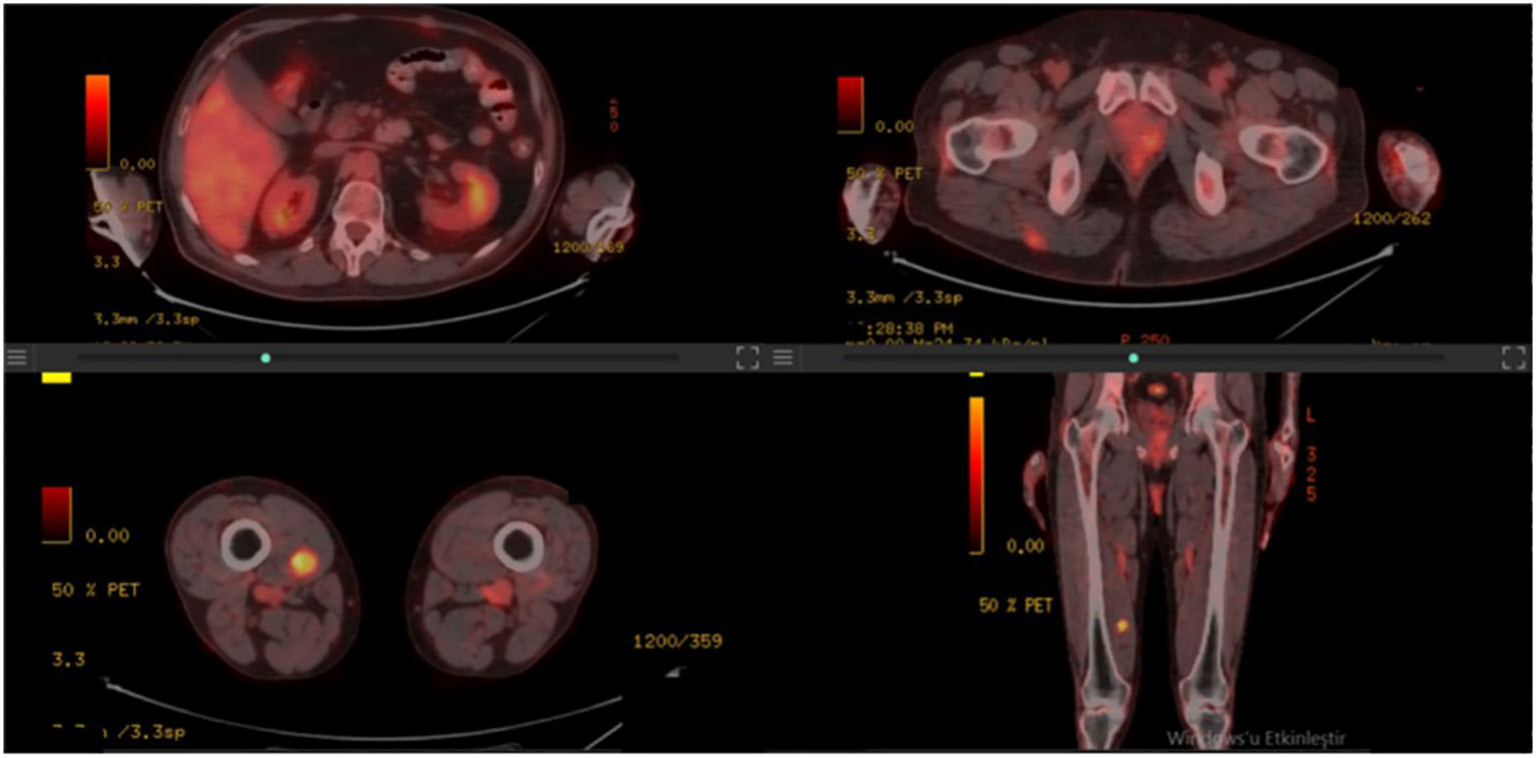
PET-CT images detecting metastatic disease.

**Figure 2:**
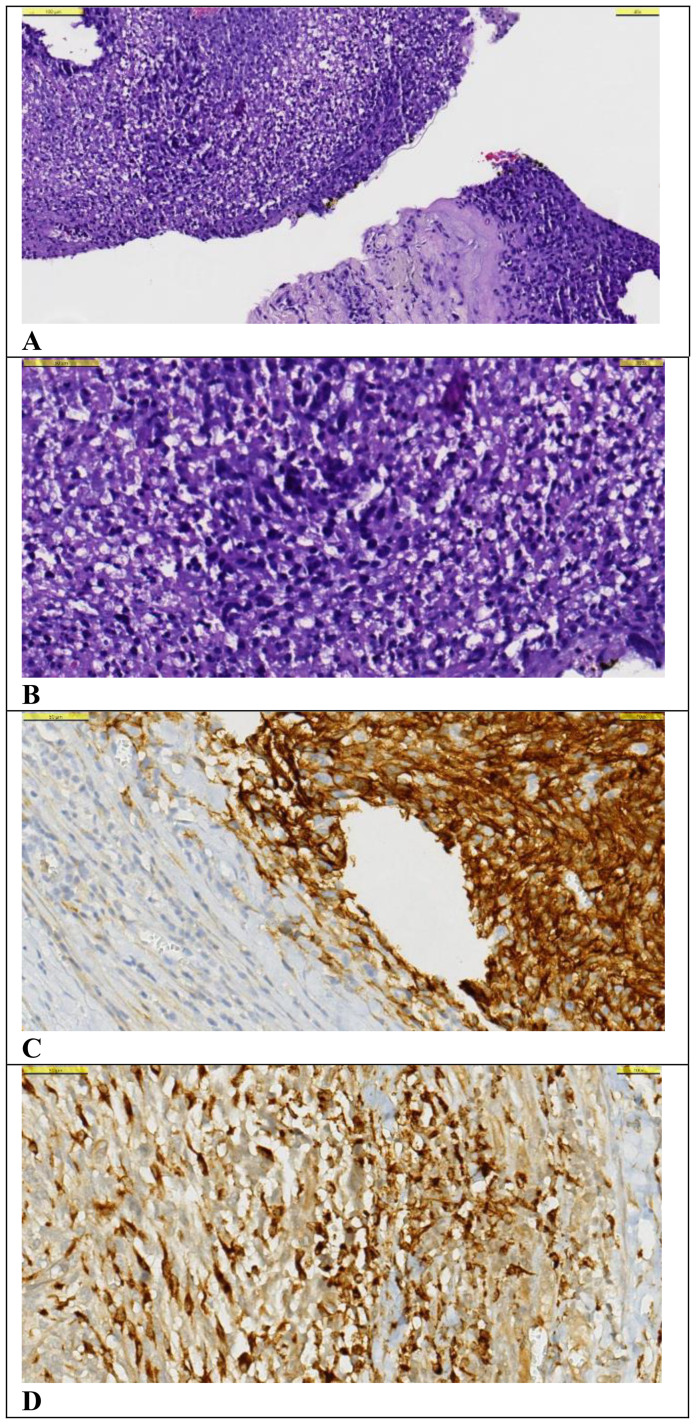
Histological examination of the tru-cut biopsy specimen from the metastatic lesion. **(A–D)**. **(A)** A small number of spindle-shaped hyperchromatic cells with narrow cytoplasm are observed on the background of necrosis and chronic inflammatory cell infiltration with Hematoxylin&Eosin (x40 magnification). **(B)** Hematoxylin&Eosin (x100 magnification). **(C)** CD10 positive staining is observed in tumor cells (x1000 magnification). **(D)** Diffuse strong cytoplasmic staining is observed in tumor cells with CD68 PGM1 (x100 magnification).

A 50-year-old male patient presented with a painful mass on the anterior chest wall. A mass measuring 60 * 60 * 35 mm located in the pectoralis major muscle was detected in the anterior part of the left third rib. The patient, who was diagnosed with undifferentiated pleomorphic sarcoma by excisional biopsy from the mass, was operated on and had negative surgical margins. In the tissue sample examined, undifferentiated pleomorphic sarcoma appeared to have infiltrated into the surrounding striated muscles, and tissue integrity was partially impaired. A 60-Gy (30 fractions, 2 Gy/fraction) three-dimensional conformal radiotherapy (3D-CRT) was applied to the post-operative tumor site to the patient who was staged by whole-body computed tomography (CT) and did not have distant metastasis. Afterward, four cycles of doxorubicin and ifosfamide were administered to the patient. The patient, who had no local recurrence or distant metastasis during a 1-year follow-up period with CT after adjuvant RT, presented again with a rapidly growing left axillary mass 15 months after RT. The Tru-Cut biopsy obtained from this mass had the same characteristics as the previous tumor. Since there were no distant metastases in the re-staging CT, the 100-mm mass was excised again with the surgical margins intact. Then, the patient was administered 60-Gy (30 fractions, 2 Gy/fraction) adjuvant RT volumetric modulated arc therapy (VMAT) at the operation site. Adjuvant chemotherapy consisting of four cycles of gemcitabine and docetaxel was continued for the patient. Another mass measuring 77 * 47 mm was seen in the posterolateral aspect of the left eighth rib of the patient 15 months after the second RT. The PET-CT staging did not reveal any far-reaching metastases (positron emission tomography). The post-operative pathology report on the third procedure on the patient showed comparable pathological findings to the first two operations, and the surgical margins were negative. Adjuvant 60-Gy (30 fractions, 2 Gy/fraction) RT was applied to the left chest wall with VMAT. PET-CT was performed 6 months after RT revealed an intense hypermetabolic lesion in the right vastus medialis muscle, mild hypermetabolic newly developed metastatic lesions in the right gluteus maximus muscle, right vastus intermedius muscle, and left rectus abdominis (**Figure 1**). On the physical examination, a 35–40-mm firm and semi-mobile mass was palpated in the rectus abdominis muscle. Other lesions could not be detected on the examination due to their deep localizations. Ultrasound (USG)-guided Tru-Cut biopsy of the mass in the right vastus medialis muscle was performed. Biopsy revealed that this mass was a metastasis of an undifferentiated pleomorphic sarcoma (**Figure 2**).

**Figure 3:**
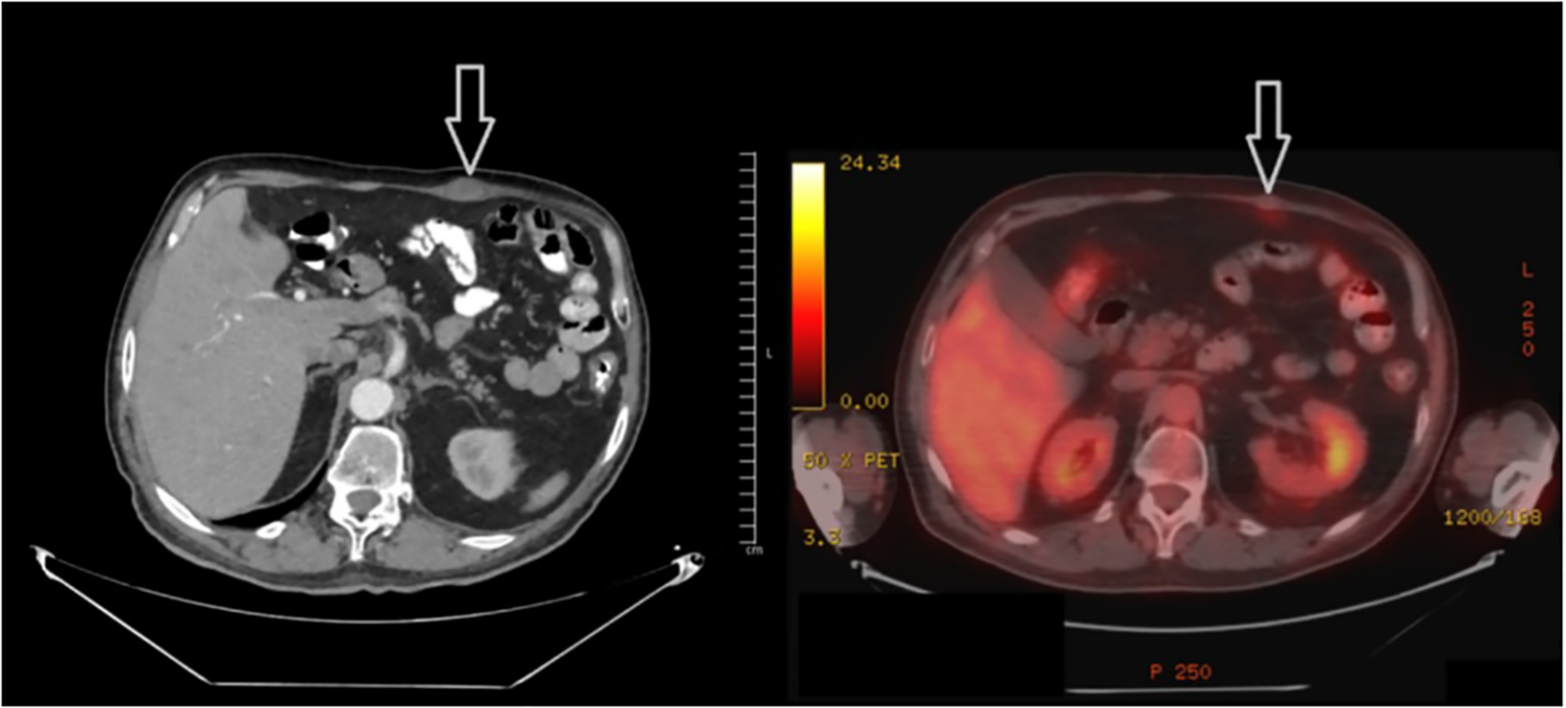
Spontaneous regression of the lesion in the left rectus abdominis muscle.

In the process of organizing and reporting the biopsy, the patient presented to the outpatient clinic again, stating that the mass in the rectus abdominis muscle shrank spontaneously. This mass, which was easily palpable in the previous examination, could not be palpated in the current one. Subsequently, it was decided to re-stage the patient by PET-CT before systemic treatment. In the PET-CT, hypermetabolic lesions in the right gluteus maximus and right vastus intermedius muscle were not detected in the current study, and the lesions in the left rectus abdominis (**Figure 3**) and right vastus intermedius muscle also regressed significantly and did not show significant pathological fluorodeoxyglucose (FDG) uptake. The patient was followed up after this circumstance was determined to be an abscopal effect. The minor hypermetabolic lesions found in the left rectus abdominis muscle and the right vastus medialis muscle were shown to have entirely regressed in the subsequent PET-CT 3 months later, and no further pathological FDG uptake focus was found in the body.

The patient, who was operated on the chest wall three times for undifferentiated pleomorphic sarcoma and was administered three times of radiotherapy and two lines of chemotherapy, tolerated these procedures very well. The patient had no new metastasis or local recurrences in the follow-up examinations and had a complete response after the abscopal effect for 48 months.

## Discussion

RT has been one of the cornerstones of cancer treatment since the beginning of the 20th century and is applied in almost half of current cancer treatments. Radiation oncology departments have been studying the killing of cancer cells by the cytotoxic effects of radiation for nearly a century (15). Today, with the developments in cancer immunology and immunotherapy, the role of the immune system in the success of cancer treatment has become a critical issue in the global cancer strategy, and the interest in the systemic effects of RT has increased (16). The anti-tumor response that occurs in the non-RT-applied area with the activation of the immune response by radiotherapy is defined as the abscopal effect. Abscopal effect is a very rare condition and was first described by Mole in 1953 by realizing the unexpected shrinkage of lesions outside the radiation field (1). It is often reported in malignant melanoma, known as immunogenic cancers (4). Apart from this, other cancer types in which the abscopal effect is observed include breast cancer, lymphoma, lung cancer, cervical cancer, renal cell cancer, and hepatocellular cancer (5–10). Today, with case reports published from all over the world, knowledge regarding the abscopal effect has increased day by day.

There are several independent mechanisms responsible for the abscopal effect. These mainly include cytokine release and T-cell activation. Following radiation exposure, the death of tumor cells and tumor surface molecules with antigenic properties are exposed in the extracellular space. T cells are activated when dendritic cells present these tumor-derived molecules, and immunogenic cell death occurs with the activation of cytotoxic T cells. In addition, cytokines such as IL-1a, IL-1b, IL-6, TNF-α, TGF-β, or IFN type I released together with radiotherapy and accompanying free oxygen radicals play a major role in the activation of T cells (17). This condition has been demonstrated in both animal and human studies. The study of Demaria et al. on rats with syngeneic mammary carcinoma demonstrated a reduction in breast cancer in these rats with the abscopal effect but no reduction in lymphoma in the same rat. Thus, the abscopal effect was revealed to be both tumor-specific and T cell-dependent (18). The findings of Suzuki et al. support the concept of immunogenic cell death after chemoradiotherapy. In a study performed on 16 patients with esophageal squamous cancer, tumor antigen-specific T-cell response was detected in 38% of the patients after chemoradiotherapy (19).

All findings support that radiotherapy not only causes cell death by DNA damage but also results in tumor response by activating the immune system with many mechanisms. Despite everything, the abscopal effect is still rare and is being investigated. With recent advances in cancer immunotherapy, there is growing interest in combining immunotherapy and radiotherapy, especially with agents that target immune checkpoints. These developments have deepened our understanding of the immune system’s role in cancer treatment and brought renewed focus to the abscopal effect and its variable timing across cases (20).

A study examining 46 cases where the abscopal effect was observed following radiotherapy found that the timing of this effect varied widely. Although the median time for the onset of the abscopal effect was reported to be 2 months (range, 0–24 months), it was noted that in a significant portion of cases, it occurred at different intervals (21). In our case, however, the abscopal effect was observed at a longer interval of 6 months post-RT. This finding places our case within the longer time range observed in the literature, highlighting that the timing of the abscopal effect can vary considerably for each patient. Abscopal effect can rarely be observed with local therapies other than RT. In breast cancer, the abscopal effect has been demonstrated not only in studies applying RT but also in cell studies combining cryoablation and immunotherapy (22). Additionally, in a case series involving three patients diagnosed with malignant melanoma, malignant melanoma with cutaneous squamous cell carcinoma (SCC), and cervical SCC, the abscopal effect was observed with metastatic tumor regression following a combination of immunotherapy and surgical intervention (23). Although there are many tumor types in which the abscopal effect is more common, it is extremely rare in malignant mesenchymal tumors and has been described in only a few cases in the literature (12–14). In our case report, a case cured by spontaneous regression from the metastatic stage as a result of the abscopal effect is presented. Additionally, the definition of the abscopal effect in our case was possible only with careful follow-up and detailed physical examination.

## Conclusion

Radiotherapy may achieve a systemic anti-tumor effect in the whole body, similar to chemotherapy, via a tumor-specific immune response. Although this condition, called the abscopal effect, is mostly reported in immunogenic tumors such as malignant melanoma, it is very rare in sarcoma cases. To determine the abscopal effect, patients should be systematically evaluated carefully, and physical examination should not be ignored.

## Data Availability

The original contributions presented in the study are included in the article/supplementary material. Further inquiries can be directed to the corresponding author.
